# Kleine-levin syndrome comorbid with obsessive compulsive disorder and personality disorder: A case report

**DOI:** 10.22088/cjim.14.1.150

**Published:** 2023

**Authors:** Pezhman Hadinezhad, Javad Setareh, Parisa Adimi naghan

**Affiliations:** 1 Psychiatry and Behavioral Sciences Research Center, Addiction Institute, Mazandaran University of Medical Sciences, Sari, Iran; 2 Department of Psychiatry, School of Medicine, Mazandaran University of Medical Sciences, Sari, Iran; 3 Department of Pulmonary and Sleep Medicine, NRITLD, Shahid Beheshti University of Medical Sciences, Tehran, Iran

**Keywords:** Kleine-Levin syndrome, Obsessive-compulsive disorder, Hypersomnia, Case report

## Abstract

**Background:**

Kleine-Levin syndrome (KLS) is a rare sleep disorder with at least two episodes of hypersomnia coincidence with at least one cognitive, eating, perceptive and disinhibited symptoms and normal inter-episodes. These symptoms are not explained by another sleep, medical, neurological, psychiatric disorders and substance or drug use.

**Case Presentation:**

Here we report a young female with personality disorder and obsessive-compulsive disorder who had KLS. Her symptoms appeared in the past 1.5 years ago, while she had an episode of hypersomnia lasting for 5 days. She had 4 attacks; each one lasted up to 2-7 days. We found that overriding KLS symptoms on underlying main psychiatric or personality disorders complicates diagnosis. All neurological examinations during episode and further investigation were in normal range.

**Conclusion:**

We suggest that taking a complete history and mental state examination in the episode and inter episode phase helps to diagnosis both KLS and comorbid psychiatric disorders.

Kleine-Levin syndrome (KLS) (also known as recurrent hypersomnia) is a rare sleep disorder with prevalence about 2 per million (1). KLS is defined by recurrent hypersomnia, at least one symptom in cognitive dysfunction, behavioral disinhibiting and eating disorder. Each episode lasts between two days to 5 weeks with normal mood and cognitive or behavioral function between episodes (2). Hypersomnia usually has a sudden onset and patients sleep about 15- 21hour per day (3). Longer durations of the first episode (more than 30 days) predicted more severe subsequent episodes and prolonged disease (1). The first episode of KLS occurred most often in autumn and winter (62%) (4). About 64-68% of patients are males and in 80% of cases onset was in during teenage. Median age of onset happens at14-15 years old. Symptoms recurred every 3.5 month and lasted in average about 8 years (1, 3-5). It was precipitated most frequently by infections (38.2%), head trauma (9%), or alcohol consumption (5.4%). Common symptoms were hypersomnia (100%), cognitive changes (96%, including a specific feeling of derealization), eating disturbances (80%), hypersexuality (43%), compulsions (29%), and depressed mood (48%). Eighty-nine percent of patients remembered an event closely associated with onset, most often infections (72%; 25% with a cold-like syndrome with fever), alcohol use (23%), sleep deprivation (22%), unusual stress (20%), travelling (10%), head trauma (9%), and marijuana use (6%) (4). Although exact etiology of this syndrome is unknown, there are some reports about relation between KLS and genetic, familial (6), immunologic (7), infectious disease, encephalopathy (8) and head trauma (9).

Hypoperfusionin hypothalamus, thalamus and basal ganglia reported in episodes but not inter episodes (10). Another study found an increase of perfusion in thalamus, caudate and lentiform nucleuses in symptomatic phase of disease (11). Pharmacological treatments such as tricyclic drugs, serotonin reuptake inhibitors, antipsychotics, anticonvulsant, antiviral, hydrocortisone, melatonin and benzodiazepines were used in these patients and indicated contradictory results (12).

Also, many psychiatric problems including anxiety, depression, disinhibition, hyperphagia, derealization, psychosis and cognitive problems were described in these patients during symptomatic phase but there are few studies about psychiatric disorders and personality disorder in asymptomatic phase of KLS. In this article, we report a patient with personality disorder and obsessive-compulsive disorder. Her symptoms began after interpersonal conflicts. 

## Case Presentation

This report has ethical code from Mazandaran University of Medical Sciences: IR. MAZUMS.REC.1400.11617. The case is a 21-year-old woman; her symptoms appeared in the past around 1.5 years ago, while having an episode of hypersomnia lasting for 5 days. She had 4 attacks; each one lasted up to 2-7days. Periods began in afternoon preceded by nearly 2 hours of cheerfulness, kindness and exaggerated love to her husband without increasing sexual desire. After that she experienced about 30 hours of immobility while she was able to hear surrounding sounds. Then she felt severe drive to sleep and slept about 16-20 hours per day, lasting up to 7 days coincidental with hyperphagia, loss of sexual interest and decreased speech. She was calm and sleepy and felt no depression or irritable mood. In each episode, she answered questions correctly, and she had normal orientation. In the last day of each episode, she became irritable and abruptly symptoms disappeared and no memory about the episode remained. The patient had an intense fear of loneliness which led to transient and unstable relationships and unstable marriage therefor she planned to divorce. The patient never had manic episode symptoms. She had 3 suicide attempts and also impulsive actions like shaving her hair. She refused using substance except for nicotine and sometimes alcohol. The patient had changed her goals and wishes in her life many times, including being a house keeper mother, soccer player, shopkeeper, immigrating to a big city, living in a rural area and so on. She sometimes was in love to her husband and sometimes hated him. Sometimes wanted to get divorce but at the final stages she got remorse. Instability in relationship had begun from school age. Since she was 14, she has obsessed to imagination of music and could not stop it. She hated number 9 and had frequent struggles because of mouth sounds of persons during eating. These symptoms made her leave places that others were eating. Also she used knocking table to stop music in her minds. These obsessions and compulsions took long for hours. All above symptoms occurred out of hyper somnolence episode. In the last 2 years, the patient has received fluoxetine 40 mg/day with little effect on recurrent thoughts. 

All neurological examinations during episode and further investigation including electroencephalography monitoring, brain MRI, endocrine evaluation e. g. thyroid function tests, serum prolactin, electrolytes, liver function test, kidney function test, ESR, CRP and other markers of inflammation were in normal range. Patient had normal cognition and perception between episodes. However, she had some reactive depressed mood without disturbance in cognitive functions for a few days in extreme duration.

## Discussion

According to international classification of sleep disorders-3 (ICSD-3) the patient fulfilled Kleine-Levin syndrome criteria based on more than 2 episodes, lasting more than 2 days and less than 5 weeks, normal cognitive and behavior between episodes. Sometimes depressed mood related to environmental problems have been observed, but the nature and phenomenology of these two were different. Hypersomnolence could not be explained by another sleep disorder or psychiatric and neurological disorders. Obsessive-compulsive disorder diagnosis is confirmed because of recurrent uncontrollable music imagination which interfered with her functions and began when she was 14 years old. It was not explained by other disorders or substance use listed in Diagnostic and Statistical Manual of mental disorders 5^th^ edition (DSM-5) (13).

The patient had unstable interpersonal relationship, impulsivity and suicidal attempts which were not in hypersomnolence period. Also, the patient had symptoms of cluster B personality disorders but did not fulfill threshold criteria for specific personality disorder. She showed interpersonal, poor impulse control, beginning at least since early adolescence, some degree of impairment in functions. Long lasting symptoms which could not be explained by another mental disorder or substance abuse confirmed the diagnosis of personality disorder according to DSM-5(13). In general OCD prevalence is 1-1.5% (14) and personality disorder about 15%(15) and Kleine-Levin syndrome about 2 per million (1) make probability of this coincidence about 6 per billion. But structured assessment of OCD and personality disorder in Kleine-Levin syndrome may define accurate result c that may reveal it is not only based on chance. 

An interesting finding was the start of symptoms with positive mood and ending with irritable mood, while between them we observed dominancy of cognitive and hallucination and also tiredness and hypersomnolence. It is in contrast to most of reports explained the beginning of KLS with negative mood and termination by positive mood (1, 3, 4, 16). Because of cognitive and other psychiatric symptoms of KLS, it is important to pay attention to other comorbidities in psychiatric and medical assessments in addition to symptoms related to this syndrome. 
